# Infrared thermography fails to visualize stimulation-induced meridian-like structures: comment by Rixin Chen and Zhimai Lv and reply from Gerhard Litscher

**DOI:** 10.1186/1475-925X-10-80

**Published:** 2011-09-11

**Authors:** Rixin Chen, Zhimai Lv, Gerhard Litscher

**Affiliations:** 1 Affiliated Hospital of Jiangxi University of Traditional Chinese Medicine, Nanchang 330006, Jiangxi Province, China; 2 The First Affiliated Hospital of Gannan Medical University, Ganzhou 341000, Jiangxi Province, China; 3 Research Unit of Biomedical Engineering in Anesthesia and Intensive Care Medicine and TCM Research Center Graz, Medical University of Graz, Auenbruggerplatz 29, 8036 Graz, Austria

## Abstract

A comment on G. Litscher: Infrared thermography fails to visualize stimulation-induced meridian-like structures. Biomed. Eng. OnLine 2005, 4:38 (15 June 2005), with a response by the author.

## Comment: Rixin Chen and Zhimai Lv

Acupuncture is an important part of traditional Chinese medicine, which contributed to the proliferation of the Chinese in the passed thousand years. However, the studies of its mechanisms began from 20th century. As the acupuncture point and the meridian being the important physiological basis of acupuncture treatment, a lot of scholars have investigated their anatomical and physiological basis. Despite all that, from the perspective of modern medicine, it could not give an accurate definition to the acupuncture point, let alone to understanding of the meridian.

We are glad to see the article, entitled "Infrared thermography fails to visualize stimulation-induced meridian-like structures", in your magazine of BioMedical Engineering OnLine [1]. The author came to the conclusion with precision testing and the strict logical analysis. However, if the design of experimental method could consider the following three aspects, the meridian-like phenomenon might be visualized.

### 1. Choosing volunteers

The acupuncture point is not a single part of the body surface; it is a functional state of certain part of the living body. The functional state is closely related to the pathological process of diseases. When a patient's condition has improved, the functional state is weakening or normalized. The so called acupuncture points on the healthy adult's surface are only parts of the body surface, while the points on the patient's surface are known as real acupuncture points [2]. The subjects selected were all healthy adults. Hence, the meridian-like structures could not be observed by stimulating on the non-specific functioning parts. On the contrary, it might be observed by choosing sick individuals as subjects.

### 2. Treatment Duration

The duration of suspended moxibustion treatment is considered to be the important facts on meridian-*Qi *excitation and its efficacy. The application of suspended moxibustion within 15 minutes is considered the stage of meridian-*Qi *excitation, while an extended period of suspended moxibustion may provide an effective treatment stage [3]. The meridian-like structures could not be observed clearly in the period of meridian-*Qi *excitation. Since the duration chose was 5-10 minutes, it was difficult to visualize the meridian-like structures. If the duration could be extended to 40 minutes, it would be helpful to observe it.

### 3. Acupuncture sites

Paging through the pre-qin period of literature, the acupuncture point is not fixed on the certain point of the body surface but a general area. There is a sensitive point in this area. If giving a proper physical stimulation to the sensitive point, such as heat or force, the meridian-*Qi *can be excited and the disease can be remedied effectively [2]. However, it was not pointed out that whether the points selected to be stimulated was the sensitive point. Therefore, it was hard to visualize the meridian-like structures.

### Conclusion

At present, many experiments related acupuncture might have been neglected the three contents in the design of the study. Therefore, the results are very different so that the effects of acupuncture or moxibustion remain elusive [4]. If we could pay an attention to the contents above, the results might be the other way.

## Reply: Gerhard Litscher

### Background of this contribution

Unfortunately the letter to the editor entitled "Infrared thermography fails to visualize stimulation-induced meridian-like structures: Comment" by Rixin Chen and Zhimai Lv contains neither data nor facts, however there are some well-known useful comments to the study design which was not performed by our group. In this context I would like to thank the editor-in-chief of Biomedical Engineering OnLine, Professor Foster, for the opportunity to summarize once again some important facts on the topic in this standard journal article, which also serves as reply.

### Bioengineering assessment of acupuncture in Graz

Basic research on high-tech acupuncture has been successfully performed in Graz since 1997 using a broad spectrum of methods. Main goal of these activities was to combine basic research on the topic of high-tech acupuncture with necessary further experimental and clinical pilot studies in China for the first time.

Acupuncture has been used as medical treatment for thousands of years. A large number of empirical data is available, but the technical quantification of effects was not possible up to now. Using electroacupuncture, needle or laser needle stimulation and modern biomedical techniques, it was possible to quantify changes in biological activities caused by acupuncture [5-15].

### Infrared thermography and meridian-like structures

**Figure 1:**
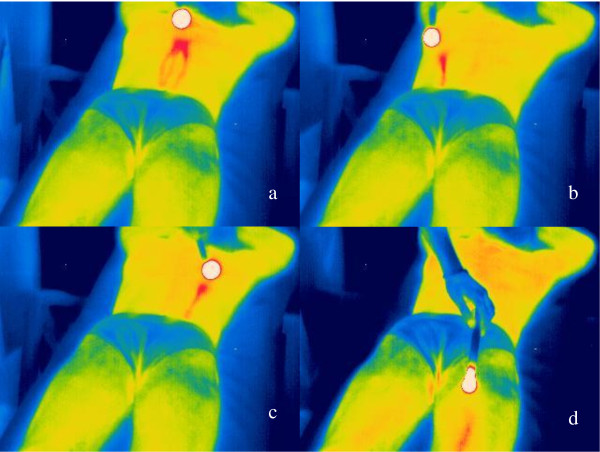
**Thermograms with reflection artifacts, but not meridians, from a volunteer**. The moxa cigar (length 20 cm, diameter 1.5 cm; Hunan, China) used for stimulation (distance about 10 cm, duration about 5 - 10 min) is visible as a circular white area (average temperature 550°C; measured with Flir ThermaCAM S65 with an energy maximum ranging from 3 - 3.5 μm [16]). Dependent on the angle of reflection, technical reflection effects visible as lines at optional parts of the body (a - d) can occur. The thermographic scale was defined from blue (25°C) to red/white (41°C). From [16].

"Thermographic methods, such as infrared cameras at wavelength ranges of 2-5 μm and 7.5-13 μm and other high-tech methods, are effective complementary methods in acupuncture research which support demystification of this treatment method. However, the validity of the method for proving meridian structures according to the view of Traditional Chinese Medicine (TCM) must be considered critically and analysed scientifically." [16] In contrast to other authors [17], we stated in our paper in 2005 that "according to current technical standings and to the method proposed by other authors [17], the visualization of energetic paths in the sense of meridians seems to be not possible using thermography" [16]. We demonstrated clearly that "after moxibustion (...) different structures appear on thermographic images of the human body which are technical artifacts and which are not identical to what are known as meridians in all textbooks of TCM" [16]. One example from a 30-year-old volunteer with reflection artifacts is shown in Figure 1.

**Figure 2:**
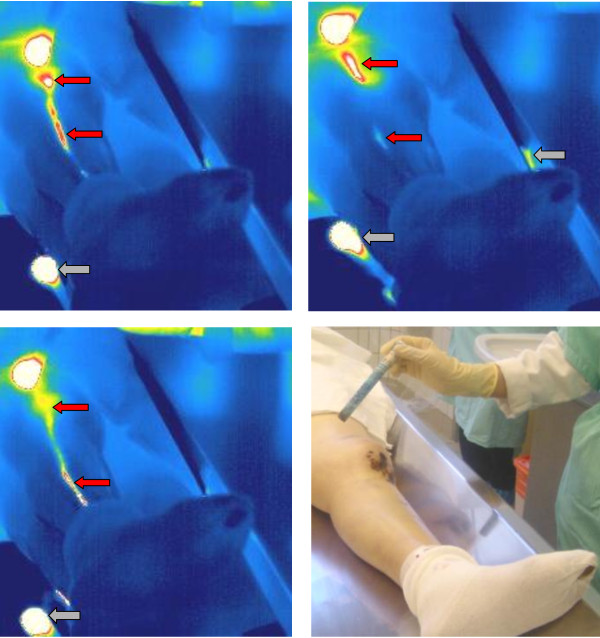
**Three thermal images (and one photograph) with artifacts in a dead subject**. The scale of the thermal images was defined from 15°C (dark blue) to 40°C (red/white). The red arrows mark the reflection at the body surface (leg), and the grey arrows those on a metal surface. Further methodological aspects see Figure 1 and references [16-18,20]. The cadaver (84 y, female) study was approved by the chairman of the ethics committee of the Medical University of Graz; see [18]. The investigation was performed 14 hrs after the determination of death. Modified from [20].

In the meantime, several other investigations on this topic were performed by our group, which are not mentioned by Chen and Lv. Together with the president of the Austrian Thermographic Society, Professor Ammer, we published a paper entitled "Visualization of equipment dependent measurement errors, but not of meridian-like channels in complementary medicine - a thermographic human cadaver study" in Thermology international in 2007 [18]. "In this study, we have visualized equipment dependent thermographic artifacts in dead subjects in whom generally no energy flow is supposed to occur. (...) These measurements demonstrate once again clearly that it is not allowed to interpret these artifacts as any kind of meridian-like structures according to TCM as other authors do." [18] One example from these measurements in dead subjects is shown in Figure 2.

### Discussion and Conclusion

Firstly, I would like to point out that Chen and Lv seem to have misunderstood the goal of our original study [12]. The main goal of this study was not to prove or disprove the existence of meridians, but to reproduce another study mentioned above [17] which claims to have found visualized proof of meridians using thermography.

"Goal of scientific methods is the demarcation of dogmas. Scientific research is based on three currently valid theories: "instrumental injection", i.e. concrete methodology, "direct perception" and "common confirmation or refutation". (see comment to [16]). We followed these guidelines exactly in our scientific publication [16] with regard to infrared analysis and meridians. With the help of modern, infrared thermographic measurement equipment, we were not able to visualize meridians (energetic paths in Traditional Chinese Medicine) using an already described and extended methodological concept." (see comment to [16])

Secondly, I would like to cite Professor John Longhurst exemplarily: "Sometimes thermograms have been thought to actually show the pathway of a weakly luminescent meridian, but such demonstrations have been inconsistent between meridians. Furthermore, there is no physical proof that such oscillations occur, that they change with disease or that they actually represent meridians. There simply is no evidence for the existence of meridians based on this theory." [19]

Thirdly, from my point of view, there is nothing that needs to be added to this statement at the moment.

## Competing interests

The authors declare that they have no competing interests.

## Authors' contributions

Chen RX and Lv ZM designed and wrote the manuscript together. GL wrote the reply. All authors read and approved the final manuscript.

## Authors' information

Chen RX, professor, the founder of Heat-sensitive Moxibustion, Affiliated Hospital of Jiangxi University of TCM, Nanchang, China.

Lv ZM, the main participant in the basic research of Heat-sensitive Moxibustion, a neurologist of the First Affiliated Hospital of Gannan Medical University, Ganzhou, China.
